# ﻿Checklist of the genus *Ridsdalea* (Rubiaceae, Gardenieae) in Vietnam with description of the new species *R.backanensis*

**DOI:** 10.3897/phytokeys.239.113017

**Published:** 2024-03-01

**Authors:** Khoa Van Phung, Leonid V. Averyanov, Tatiana V. Maisak, Ludmila I. Krupkina, Hai Xuan Cao, Cuong Huu Nguyen

**Affiliations:** 1 Vietnam National University of Forestry, Xuan Mai, Chuong My, Ha Noi, Vietnam Vietnam National University of Forestry Ha Noi Vietnam; 2 Komarov Botanical Institute Russian Academy of Sciences, Prof. Popov str., 2, St. Petersburg, 197376, Russia Komarov Botanical Institute Russian Academy of Sciences St. Petersburg Russia

**Keywords:** Ba Be National Park, Bac Kan Province, endemism, flora of Vietnam, new species, plant diversity, plant taxonomy, *Rothmannia*

## Abstract

The paper provides an identification key and an annotated list of all six species of the genus *Ridsdalea* J.T.Pereira & K.M.Wong (Rubiaceae, Gardenieae) recorded in the flora of Vietnam, along with data on their valid names, synonyms, types, and distribution. A new species, *R.backanensis*, discovered in the limestone area of Bac Kan Province (northern Vietnam), is described and illustrated. Detailed data on its characters, ecology, distribution, phenology, preliminary IUCN conservation status, and taxonomical notes are given. The newly discovered species is potentially interesting for cultivation as an ornamental tree that may be effectively used in garden and urban green architecture.

## ﻿Introduction

The genus *Ridsdalea* J.T.Pereira & K.M.Wong (in Wong and Pereira 2016: 42) was segregated from *Rothmannia* Thunb. (Thunberg 1776: 65) mainly because its corolla lobes are contorted to the left, whereas the lobes in *Rothmannia* s.str. are contorted to the right. Both genera are closely related and belong to the tribe Gardenieae, subfamily Ixoroideae of the coffee family (Rubiaceae) (Wong and Pereira 2016). While species of *Rothmannia* are distributed in Africa, representatives of the closely related *Ridsdalea* inhabit wide areas in the tropics of Southeast mainland Asia and Malesia (Wong and Pereira 2016).

The twelve genera of the tribe Gardenieae occurring in Vietnam, namely *Aidia* Lour., *Aidiopsis* Tirveng., *Alleizettella* Pit., *Brachytome* Hook.f., *Dioecrescis* Tirveng., *Duperrea* Pierre ex Pit., *Gardenia* J.Ellis, *Kailarsenia* Tirveng., *Oxyceros* Lour., *Randia* L., *Rubovietnamia* Tirveng., and *Vidalasia* Tirveng. (Pham 2000; Tran 2005), are well segregated from each other morphologically and well-supported by molecular data (Robbrecht and Manen 2006; Bremer 2009; Mouly et al. 2014). Besides left-contorted corolla lobes, typical diagnostic characteristics of the genus *Ridsdalea* are such features as large shrub or tree habit; unarmed shoots with opposite or verticillate leaves (in groups of 3); interpetiolar stipules without distinct venation; terminal or pseudoaxillary, 1–7-flowered, cymose inflorescences; bisexual flowers; 2-celled ovaries with axile placentation; and, large, baccate, many-seeded fruits (Chen and Taylor 2011; Wong and Pereira 2016).

All species of *Ridsdalea* are rather large shrubs or small, medium, or large trees scattered in lowland or submontane woods on soils derived from various parental rocks. Some species prefer exclusively alluvial soils in limestone areas and often inhabit rocky, steep slopes of rocky karstic hills.

Twenty-eight species of *Ridsdalea* distributed in Indochina and Malesia were recognized in a comprehensive monograph of the genus (Wong and Pereira 2016, POWO 2023), and a few species were added by recent studies in Thailand (Kwanjai et al. 2018) and the Philippines (Bustamante and Pelser 2022). In Vietnam, five species were previously reported (Zhang et al. 2007; Bui and Nguyen 2015; Ton et al. 2019), which are listed below together with the newly discovered species.

During botanical fieldwork in November 2022 in limestone areas of Bac Kan Province in northern Vietnam, we collected several specimens sharing the above-mentioned generic characteristics of *Ridsdalea*, but morphologically different from all species hitherto known by a number of unique characteristics. After consulting relevant literature (Thunberg 1776; Pitard 1923; Tirvengadum 1983; Shui et al. 2003; Zhang et al. 2007; Bui and Nguyen. 2015; Wong and Pereira 2016; Kwanjai et al. 2018; Ton et al. 2019; Bustamante and Pelser 2022), as well as examining available herbarium specimens stored at HN, HNPI, HNU, K, LE, P, VNF, and VNM, we identified our plants as a new species on the basis of the morphological features indicated in the identification key and diagnosis, and protologue. With the new data reported here, the number of species of the genus *Ridsdalea* in the flora of Vietnam reaches six. The paper aims at formally recognizing the novelty, with a key to the Vietnamese species and a briefly annotated checklist.

## ﻿Materials and methods

All collected and studied herbarium specimens of the newly discovered species are presently stored in the herbaria of the Vietnam National University of Forestry (VNF) and the Komarov Botanical Institute of the Russian Academy of Sciences (LE). Color photos of plants were taken in natural habitats. Morphological observations and measurements were made on living plants, dried specimens, and alcohol-preserved materials. Morphological characters were described using the terminology proposed by Harris and Harris (2006), Hickey and King (2000), and Beentje (2016). Institutes where studied specimens are kept are indicated by their internationally accepted herbarium acronyms (Thiers 2024).

## ﻿Taxonomic treatment

### ﻿*Ridsdalea* species in the flora of Vietnam

#### ﻿ Ridsdalea

Taxon classificationPlantaeGentianalesRubiaceae

J.T.Pereira & K.M.Wong (in Wong and Pereira 2016: 42).

4F1000AE-F628-55CD-9940-2EB2F4173329

##### Type.

*Ridsdaleagrandis* (Korth.) J.T.Pereira (in Wong and Pereira 2016: 46). ≡ *Gardeniagrandis* Korth. (Korthals 1851: 191).

##### Description.

Large shrubs to small, medium or large trees with unarmed trunk and branches. Branches opposite and decussate on the trunk, sympodial in development. Leaves opposite or verticillate in groups of 2–3, each trifoliate group based on a distal node with only one leaf normally developed and the proximal node with a pair of normal leaves; leaves petiolate to subsessile, axils of the midrib, secondary veins, and sometimes other vein junctions frequently with domatia on the abaxial leaf surface; margin entire. Stipules interpetiolar, without distinct venation, free or hardly fused at the base, persistent to caducous, inner surface hairy with colleters restricted to 1–several rows. Inflorescence terminal or pseudoaxillary (terminal in origin, later appearing lateral because of displacement to one side during sympodial branch development), distinctly cymose, several-flowered or sometimes reduced to a 1-flowered structure, distinctly pedunculate to subsessile. Flowers bisexual, pedicellate or sometimes subsessile, small or large with corolla tube less than 15 cm long. Calyx 5–8(–10)-lobed, colleters present in small groups on the inner surface, glabrous or glabrescent to hairy on the outside. Corolla commonly 5-, rarely 6–8-merous; hypocrateriform or campanulate; the tube white or white with reddish purple speckles or blotches inside, the outer and inner surface glabrous or hairy; lobes contorted to the left in the flower bud; stamens attached at the upper part of the corolla tube, anthers sessile or subsessile, linear or narrowly lanceolate; style shorter than, as long as, or slightly exceeding the corolla tube, mostly glabrous; stigma clavate to fusiform, 2-lobed, smooth to somewhat ribbed; ovary 2-celled; ovules many; placentation axile. Mature fruits baccate, indehiscent, globose or broadly ellipsoid, 2–5 cm across, 2-locular, outer surface smooth. Seeds many, immersed in a dark brown pulp-like placental tissue (Chen and Taylor 2011; Wong and Pereira 2016).

32 species in Myanmar, SW China, Thailand, Laos, Cambodia, Vietnam, Malacca Peninsula. Indonesia, Philippines, New Guinea. In Vietnam 6 species (2 endemic, found in Bac Kan and Lam Dong provinces).

### ﻿Key for the identification of *Ridsdalea* species in the flora of Vietnam

**Table:** 

1	Flowers solitary in leaf axils, almost sessile; peduncle 0.5–1 mm long	***R.kampucheana***
–	Inflorescences terminal or axillary (pseudoaxillary), 1–7-flowered; on distinct peduncle 7–15 mm long	**2**
2	Leaf blade tapering gradually to acute or hardly acuminate apex	***R.eucodon***
–	Leaf blade distinctly acuminate with suddenly narrowing apex	**3**
3	Inflorescence usually 4–7 flowered; corolla tube narrowly conoid, dilating gradually in distal direction, near the apex 1–1.3 cm in diameter; throat 3.5–4 cm in diameter	***R.vietnamensis***
–	Inflorescence usually 1–5 flowered; corolla tube broadly campanulate, dilating suddenly near the base, near the apex 2.5–3.5 cm in diameter; throat 4.5–5.5 cm in diameter	**4**
4	Inflorescence usually 3–5 flowered; calyx tube 3–5 mm long, calyx lobes broadly triangular, less than 3 mm long	***R.wittii***
–	Inflorescence usually 1–3 flowered; calyx tube 1.2–2.5 mm long, calyx lobes linear oblong or subulate, 6.5–16 mm long	**5**
5	Inflorescence uniflorous; pedicels 2–2.5 cm long; calyx tube funnel-shaped, 2–2.5 mm long, calyx lobes linear oblong, 14–16 mm long; corolla outside glabrous; anthers linear, ca. 1 mm wide	***R.daweishanensis***
–	Inflorescence 1–3 flowered; pedicels 3.5–7 mm long; calyx tube cup-shaped, 1.2–2.2 mm long, calyx lobes subulate, 6.5–11 mm long; corolla outside shortly sparsely hairy; anthers oblanceolate, 2–3 mm wide	***R.backanensis***

#### ﻿ Ridsdalea backanensis

Taxon classificationPlantaeGentianalesRubiaceae

C.H.Nguyen & Aver. sp. nov.

C9D131B8-271D-5B2F-9561-4545DB4EEE1E

urn:lsid:ipni.org:names:77337445-1

Figs 1 , 2

##### Diagnosis.

*Ridsdaleabackanensis* differs from *R.daweishanensis* mainly in the smaller leaves, 7–11 cm long (vs. leaves 10–14 cm long in *R.daweishanensis*), the 1–3-flowered inflorescence (vs. inflorescences uniflorous), the much shorter pedicels, 3.5–7 mm long (vs. pedicels 20–25 mm long), the shorter subulate calyx lobes, 6.5–11 mm long (vs. calyx lobes narrowly oblanceolate, 14–16 mm long), the corolla outside sparsely hairy (vs. corolla outside glabrous), and the oblanceolate anthers, 2–3 mm wide (vs. anthers linear, about 1.5 mm wide).

**Figure 1.**
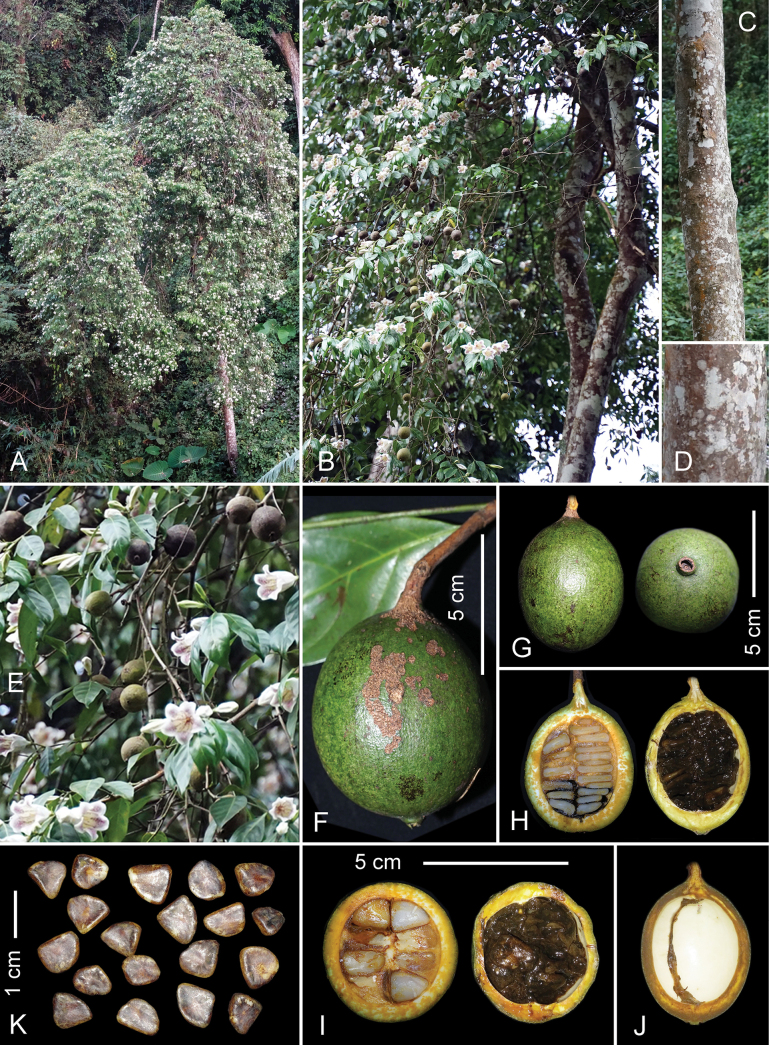
*Ridsdaleabackanensis***A** mature tree in natural habitat **B** part of crown with two main trunks **C** trunk of mature tree **D** bark at DBH **E** flowering and fruiting branches **F, G** ripe fruits **H** ripe fruits, sagittal section **I** ripe fruit, cross section **J** fruit, sagittal section, seeds removed **K** ripe seeds. Photos by C.H. Nguyen (**A–E**) and L. Averyanov (**F–K**) from plant used for preparation of the paratype voucher specimens (*AL 1680*), photo-correction, and design by L. Averyanov and T. Maisak.

##### Type.

Vietnam, Bac Kan Province: Ba Be District, Ba Be National Park, dry evergreen broad-leaved old secondary forest with domination of *Burretiodendronhsienmu* Chun & F.C.How, *Streblusasper* Lour., and *Arengapinnata* (Wurmb) Merr., on crystalline highly eroded rocky limestone near the boat station at elev. 250–300 m a.s.l., tree 10–15 m on shady rocky steep slope, not common, 18 November 2022, *Nguyen Huu Cuong*, *Cao Xuan Hai*, *L. Averyanov*, *T. Maisak*, *AL 1682* (Holotype: VNF NHC20221118006!; Isotypes: LE LE01169974! https://en.herbariumle.ru/?t=occ&id=160358, LE LE01169975! https://en.herbariumle.ru/?t=occ&id=160361, LE LE01169983! https://en.herbariumle.ru/?t=occ&id=163681; photos of living material made before preparation of type herbarium specimens: LE LE01123672! https://en.herbariumle.ru/?t=occ&id=155829, LE LE01123671! https://en.herbariumle.ru/?t=occ&id=155828).

##### Etymology.

The species is named after the area of origin (Bac Kan Province, northeastern Vietnam).

##### Description.

**Figure 2.**
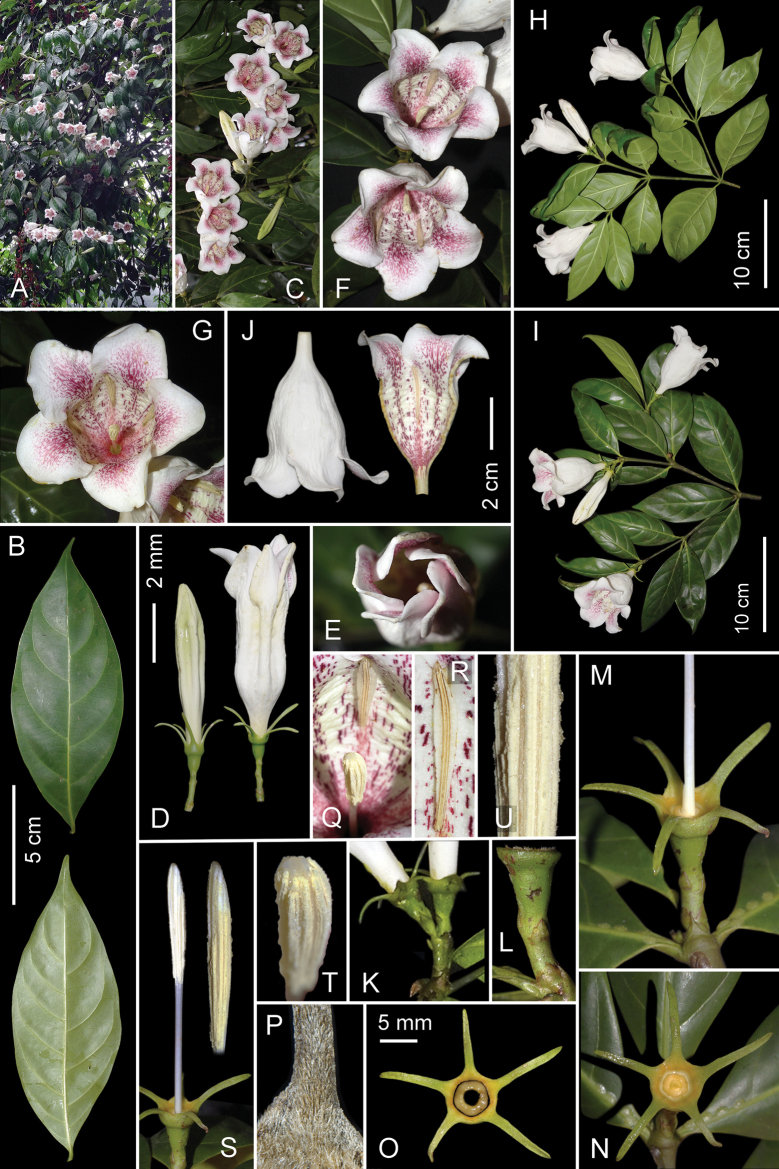
*Ridsdaleabackanensis***A** part of crown of flowering tree in natural habitat **B** leaves, adaxial and abaxial surface **C** flowering branches **D** flower bud and opening flower, side view **E** Opening flower, frontal view **F, G** flowers, frontal view **H** flowering branch showing leaves from abaxial side **I** flowering branch showing leaves from adaxial side **J** corolla, sagittal section outside and inside **K** peduncle, pedicels and calyxes, side view **L** peduncle and pedicel, side view **M** peduncle, pedicel, calyx and base of style, side view, corolla removed **N** calyx, frontal view, corolla removed **O** separated, upper part of calyx tube, calyx lobes, and nectary disc, frontal view **P** indumentum of abaxial surface of calyx on dry specimen **Q** portion of adaxial surface of corolla, sessile stamen and apical part of stigma **R** stamen **S** pedicel, calyx and pistil with magnified stigma, side view **T** apical part of stigma **U** middle part of stigma Photos by L. Averyanov (**A, C, F–K, P**) and C.H. Nguyen (**B, D, E, L–O, Q–U**) from plant used for preparation of the type voucher specimens (*AL 1682*), photo-correction and design by L. Averyanov and T. Maisak.

Evergreen tree, (4)5–20(22) m tall with obscurely ovoid crown and solitary trunk to 30(35) cm DBH. Bark lenticellate, smooth greenish gray. Young twigs dichotomous, slightly flattened or angular, older ones terete, glabrous. Leaves entirely glabrous, shortly petiolate, opposite, or in whorls of 3; petiole obscurely half circular in cross section, adaxially shallowly grooved, (1.5)2–5(7) mm long, (2.5)3–5(5.5) mm wide; stipules interpetiolar, broadly triangular (1)1.5–2.2(2.5) mm long, (2.5)3–5(5.5) mm wide, broad and slightly fused at the base, shortly acuminate at apex, adaxially densely hairy with short hairs; leaf blade chartaceous to slightly coriaceous, narrowly ovate or narrowly obscurely rhomboid, (7)8–10(11) cm long, (3)3.5–5(5.5) cm wide, tapering from the middle to cuneate base and to shortly acuminate, obtuse apex; both surfaces dark green, glossy, median vein shallowly canaliculate on adaxial side, prominent abaxially, secondary (lateral) veins (4)5–6(7) pairs. Inflorescence cymose, 1–3-flowered, terminal or pseudoaxillary; peduncle (1.5)2–8(9) mm long, glabrous or sparsely hairy with stiff adpressed hairs, with 1–2 very small adpressed triangular alternate bracts, (1)1.2–1.8(2) mm long and wide. Pedicels (3.5)4–6(7) mm long, sparsely hairy with sericeous adpressed hairs, with 1(2) bracteoles; bracteoles alternate, triangular, (1.5)2(2.5) mm long and wide, acute to apiculate, glabrous outside, densely hairy with stiff hairs inside. Calyx green, deciduous; calyx tube and lobes glabrous or sparsely hairy with sericeous adpressed hairs outside, inside densely hairy with stiff, yellowish gray hairs; calyx tube (1.2)1.5–2(2.2) mm long, (4)4.5–6(7) mm in diameter; free calyx lobes recurved, subulate, tapering from base to obtuse apex, (6.5)7–10(11) mm long, (0.7)0.8–1.2(1.4) mm wide, without distinct veins. Corolla campanulate, outside pure white, sparsely hairy with small sericeous adpressed hairs, inside glabrous, finely transversally folded (seen when fresh, Fig. 2Q), white with light yellow tint and many purple speckles and longitudinal streaks, lasting 3–4 days, (5)5.5–6(6.5) cm long, (2.5)2.8–3.2(3.5) cm in diameter at the middle, abruptly contracted at the base into a short, narrow, 2.5(3–3.5(4) mm wide tube over a length of (7)8–10(11) mm; corolla lobes 5, overlapping to left, glabrous or sparsely hairy with small sericeous adpressed hairs outside, glabrous inside, triangular broadly ovate, (1.2)1.4–1.6(1.8) cm long and wide, blunt to obtuse at apex, white outside, purple-mottled inside, recurved. Stamens 5, alternate to corolla lobes, subsessile, inserted on the upper part of the corolla tube; anthers oblanceolate, narrowing and obtuse at the apex, (1.8)1.9–2.1(2.2) cm long, (2)2.2–2.5(3) mm wide, longitudinally dehiscent by linear thecae. Disc prominent, annular, (2.4)2.5–2.8(3) mm in diameter, glossy yellow. Style and stigma erect, (4)4.5(5) cm long; style white, terete, about 2.5–3 cm long, 0.8–1 mm in diameter; stigmatic part pale yellow, narrowly ellipsoid, longitudinally ribbed, 1.5–2 cm long, 2–2.5 mm in diameter; ovary inferior, unilocular, with many ovules in two rows. Fruit berry-like, indehiscent, dark green to dull olive-brownish, glabrous, globular to broadly ellipsoid or ovoid, (4.5)5–7(7.5) cm long, (3.5)4–4.5(5) cm in diameter, with semi-woody wall; seeds (15)20–55(65), white, flattened, obscurely triangular to lenticular, (7)8–12(13) mm long and wide, (2.5)3–4(4.5) mm thick, sunk in a dark brown or black pulpy matrix.

##### Distribution.

The new species is only known from Nam Mau Commune, Ba Be District (in the limits of the territory of Ba Be National Park), Bac Kan Province, northeastern Vietnam. It is likely endemic to a very limited area of karstic rocky limestone associated with Ba Be National Park. This location lies near the northeast border of the generic area in Indochina.

##### Habitat.

Primary and old secondary dry evergreen broad-leaved forest with domination of *Burretiodendronhsienmu* Chun & F.C.How, *Streblusasper* Lour., and *Arengapinnata* (Wurmb) Merr. on crystalline highly eroded rocky limestone at elevations of 150–300 m a.s.l., commonly on shady rocky steep slopes or in rocky valleys between limestone hills. Occasional.

##### Phenology.

Flowering from November to December, fruiting from December to January next year.

##### Preliminary IUCN conservation status.

Only two subpopulations were discovered on rocky karstic limestone in the middle part of Ba Be National Park, with few mature trees. The species meets the following conditions (Red List IUCN 2023): extent of occurrence (EOO) < 100 km^2^ (B1) and area of occupancy (AOO) < 10 km^2^ (B2) with one known location (a), and continuing decline of quality of habitat (b). We estimate that in the two subpopulations currently known, fewer than 50 mature individuals occur. These conditions identify the conservation status of the new species as globally Critically Endangered CR B1ab(iii) + B2ab(iii) As a result, *Ridsdaleabackanensis* deserves the highest priority for conservation.

##### Notes.

The new species is most similar in its morphology to *Ridsdaleadaweishanensis* described from SE Yunnan (Maguan & Hekou counties) and also reported for NW Vietnam, Lao Cai Province (Zhang et al. 2007; Chen and Taylor 2011; Bui and Nguyen 2015). The new species differs from *R.daweishanensis* in its height to 22 m tall (vs. height of tree to 15 m), the leaf blades 7–11 cm long and 3–5.5 cm wide (vs. 10–14 cm long, 4–5 cm wide), the 1–3-flowered inflorescences (vs. inflorescences uniflorous), the 3.5–7 mm long pedicels (vs. pedicels 2–2.5 cm long), the free subulate calyx lobes, tapering from base to apex and broadest at the base, 6.5–11 mm long, without distinct veins (vs. calyx lobes narrowly oblanceolate, broadest above the middle, 14–16 mm long, 1-nerved), the 5–6.5 cm long corolla, sparsely hairy with small sericeous adpressed hairs outside and glabrous inside, contracted at the base into a narrow tube, 7–11 mm long, 2.5–4 mm in diameter (vs. corolla 5–5.5 cm long, entirely glabrous, contracted at the base into 6–7 mm long tube, 5 mm in diameter), the corolla lobes glabrous or sparsely hairy with small sericeous adpressed hairs outside and glabrous inside, 1.2–1.8 cm wide (vs. corolla lobes entirely glabrous, 2.2–2.3 cm wide), the oblanceolate, 2–3 mm wide anthers (vs. anthers linear, about 1.5 mm wide), and the globular to broadly ellipsoid or ovoid fruits, containing 15–65 seeds (vs. fruits globular, containing fewer than 20 seeds).

The newly discovered plant species will doubtless be of considerable interest for cultivation as an ornamental, since it is a nice-flowering tree that may be effectively used for gardens and urban green architecture.

##### Additional material studied

**(*paratypes*).** Vietnam, Bac Kan Province: Ba Be District, central part of Ba Be National Park, dry evergreen broad-leaved old secondary forest with domination of *Burretiodendronhsienmu*, *Streblusasper* and *Arengapinnata* on crystalline highly eroded rocky limestone along Ba Be River at elev. c. 250 m a.s.l., tree 10–15 m tall on rocky slope in humid valley between limestone hills, occasional, 17 November 2022, *Nguyen Huu Cuong*, *Cao Xuan Hai*, *L. Averyanov*, *T. Maisak*, *AL 1680* (LE LE01169964 https://en.herbariumle.ru/?t=occ&id=163682, VNF-NHC 20221117004, photos of living material made before preparation of voucher herbarium specimens: LE LE01123669 https://en.herbariumle.ru/?t=occ&id=155826). Vietnam, Bac Kan Province, Ba Be District, Ba Be National Park, dry evergreen broad-leaved secondary forest on steep rocky slopes composed by white crystalline marble-like highly eroded limestone near Dau Dang Waterfall of Nang River around point 22°27'09"N, 105°34'16"E, at elevation of about 150 m a.s.l., tree about 5 m tall on shady steep rocky slope, young flower buds green, locally common, 20 October 2023, *Nguyen Huu Cuong*, *Chu Ngoc Quan*, *L. Averyanov*, *Nguyen Van Ly*, *T. Maisak*, *AL2330* (LE LE01253796 https://en.herbariumle.ru/?t=occ&id=212995, photos of living material made before preparation of voucher herbarium specimens: LE LE01124442 https://en.herbariumle.ru/?t=occ&id=207144, VNF). Vietnam, Bac Kan Province, Ba Be District, Ba Be National Park, dry evergreen broad-leaved secondary forest on steep rocky slopes composed by white crystalline marble-like highly eroded limestone near Dau Dang Waterfall of Nang River around point 22°27'09"N, 105°34'16"E, at elevation of about 150 m a.s.l., tree about 8 m tall on shady steep rocky slope, fruits broadly ellipsoid, dirty green to almost black, locally common, 20 October 2023, *Nguyen Huu Cuong*, *Chu Ngoc Quan*, *L. Averyanov*, *Nguyen Van Ly*, *T. Maisak*, *AL2331* (LE LE01253792 https://en.herbariumle.ru/?t=occ&id=212991, LE LE01253793 https://en.herbariumle.ru/?t=occ&id=212992, photos of living material made before preparation of voucher herbarium specimens: LE LE01124443 https://en.herbariumle.ru/?t=occ&id=207145, VNF). Vietnam, Bac Kan Province, Ba Be District, Ba Be National Park, dry evergreen broad-leaved secondary forest on very steep rocky slopes composed by white crystalline marble-like highly eroded limestone along lake shore on point 22°24'58"N, 105°36'48"E, at elevation of about 250 m a.s.l., tree about 10 m tall on very steep rocky slope, not common, 29 October 2023, *Nguyen Huu Cuong*, *Chu Ngoc Quan*, *L. Averyanov*, *Nguyen Van Ly*, *T. Maisak*, *AL2369* (LE LE01253794 https://en.herbariumle.ru/?t=occ&id=212993, LE LE01253795 https://en.herbariumle.ru/?t=occ&id=212994, photos of living material made before preparation of voucher herbarium specimens: LE LE01124480 https://en.herbariumle.ru/?t=occ&id=207183, VNF).

#### ﻿ Ridsdalea daweishanensis

Taxon classificationPlantaeGentianalesRubiaceae

(Y.M.Shui & W.H.Chen) J.T.Pereira (in Wong and Pereira 2016: 45).

FAA807C5-B824-5B66-B9A3-5FC386317F3D

≡ Rothmannia daweishanensis Y.M.Shui & W.H.Chen (Shui et al. 2003: 322, Zhang et al. 2007: 92). Type. China. Yunnan: Maguan, Gulinqing, Woody Station nearby Nanxi Community of Hekou, *Shui et al. 14496* (Holotype: KUN; Isotypes: KUN, MO).

##### Distribution.

S China (SE Yunnan), NW Vietnam (Lang Son Province).

#### ﻿ Ridsdalea eucodon

Taxon classificationPlantaeGentianalesRubiaceae

(K.Schum.) J.T.Pereira (in Wong and Pereira 2016: 45).

DD61B065-710F-52CE-8B00-6D16B0FDF7E0

≡ Randia eucodon K.Schum. (in Schmidt 1902: 333). Type. Thailand. Koh Chang, *Schmidt 717* (Holotype: C; Isotype: K).
= Randia exaltata Griff. var. griffithiana Pierre ex Pit. (Pitard 1923: 246). Lectotype. Vietnam. Bienhoa: Bao Chiang, *Pierre 1625* (Lectotype: P P00199261 designated by Wong and Pereira 2016: 45; Isolectotypes: K, NY, P P00199260).

##### Distribution.

S Thailand, S Vietnam (Dong Nai and Khanh Hoa provinces).

#### ﻿ Ridsdalea kampucheana

Taxon classificationPlantaeGentianalesRubiaceae

(Tirveng.) J.T.Pereira (in Wong and Pereira 2016: 46).

19A17972-CB3A-592D-AB05-F65FBA08CB8B

≡ Rothmannia kampucheana Tirveng. (Tirvengadum 1983: 466). Type. Cambodia. Nord de Kampot, 5 Feb 1928, *Poilane 14676* (Holotype: P).

##### Distribution.

SW Cambodia, S Vietnam (An Giang and Kien Giang provinces).

#### ﻿ Ridsdalea vietnamensis

Taxon classificationPlantaeGentianalesRubiaceae

(Tirveng.) J.T.Pereira (in Wong and Pereira 2016: 43).

CBF67D7F-EDFE-5619-AED1-A52C72FDE184

≡ Rothmannia vietnamensis Tirveng. (Tirvengadum 1983: 469). Type. Vietnam. Annam, col de Braian, Haut Douai, 14 Feb (rec. Jul) 1935, *Poilane 24313* (Holotype: P).

##### Distribution.

S Vietnam (Lam Dong Province).

#### ﻿ Ridsdalea wittii

Taxon classificationPlantaeGentianalesRubiaceae

(Craib) J.T.Pereira (in Wong and Pereira 2016: 53).

4464F403-074D-5196-B797-D762FFEF2F87

≡ Randia wittii Craib (Craib 1911: 392). Type. Thailand. Lower Siam: Korat, in dry deciduous jungle, 60 m, *Witt s.n.* (not located).

##### Distribution.

NE Thailand, Laos, S Vietnam (Khanh Hoa Province).

## Supplementary Material

XML Treatment for Ridsdalea

XML Treatment for Ridsdalea backanensis

XML Treatment for Ridsdalea daweishanensis

XML Treatment for Ridsdalea eucodon

XML Treatment for Ridsdalea kampucheana

XML Treatment for Ridsdalea vietnamensis

XML Treatment for Ridsdalea wittii

## ﻿Additional information

### Author ORCIDs

Khoa Van Phung https://orcid.org/0000-0002-9133-166X

Leonid V. Averyanov https://orcid.org/0000-0001-8031-2925

Tatiana V. Maisak https://orcid.org/0000-0001-5919-6755

Ludmila I. Krupkina https://orcid.org/0000-0003-0391-533X

Hai Xuan Cao https://orcid.org/0000-0002-9396-2879

Cuong Huu Nguyen https://orcid.org/0000-0003-2281-741X

### Data availability

All of the data that support the findings of this study are available in the main text.
